# Sociodemographic and economic factors are associated with weight gain between before and after cancer diagnosis: results from the prospective population-based NutriNet-Santé cohort

**DOI:** 10.18632/oncotarget.17676

**Published:** 2017-05-08

**Authors:** Philippine Fassier, Laurent Zelek, Patrick Bachmann, Marina Touillaud, Nathalie Druesne-Pecollo, Valentin Partula, Serge Hercberg, Pilar Galan, Patrice Cohen, Hélène Hoarau, Paule Latino-Martel, Bernard Srour, Rebeca Gonzalez, Mélanie Deschasaux, Mathilde Touvier

**Affiliations:** ^1^ Nutritional Epidemiology Research Team (EREN): Inserm U1153, Inra U1125, Cnam, Paris 5, 7 and 13 Universities, Sorbonne Paris Cité Epidemiology and Statistics Research Center, F-93017, Bobigny, France; ^2^ Oncology Department, Avicenne Hospital, F-93017, Bobigny, France; ^3^ Cancer, Environment and Nutrition Unit, Léon Bérard Center, F-69000, Lyon, France; ^4^ Public Health Department, Avicenne Hospital, F-93017, Bobigny, France; ^5^ Sociology Department, University of Rouen, DySola, EA 4701, F-76821, Rouen, France

**Keywords:** weight gain, weight loss, cancer diagnosis, breast cancer, socio-demographic factors

## Abstract

**Purpose:**

While many cancer patients are affected by weight loss, others tend to gain weight, which may impact prognosis and risk of recurrence and of second cancer. The aim of this prospective study was to investigate weight variation between before and after cancer diagnosis and socio-demographic, economic, lifestyle and clinical factors associated with moderate-to-severe weight gain.

**Methods:**

1051 incident cases of first primary cancer were diagnosed in the NutriNet-Santé cohort between 2009 and 2015. Weight was prospectively collected every 6 months since subjects’ inclusion (i.e. an average of 2y before diagnosis). Mean weights before and after cancer diagnosis were compared with paired Student's t-test. Factors associated with moderate-to-severe weight gain (≥5% of initial weight) were investigated by age and sex-adjusted logistic regression.

**Results:**

Weight loss was observed in men (-3.54±4.39kg in those who lost weight, p=0.0002) and in colorectal cancer patients (-3.94±4.40kg, p=0.001). Weight gain was observed in breast and skin cancers (2.83±3.21kg, p=0.04, and 2.96±2.75kg, p=0.04 respectively). Women (OR=1.75[1.06-2.87],p=0.03), younger patients (2.44[1.51-3.70],p<0.0001), those with lower income (OR=1.30[1.01-1.72],p-trend=0.007), lower education (OR=1.32[1.03-2.70],p-trend=0.03), excess weight before diagnosis (OR=1.64[1.12-2.42],p=0.01), lower physical activity (OR=1.28[1.01-1.64],p=0.04) and those who stopped smoking (OR=4.31[1.99-9.35],p=0.005]) were more likely to gain weight. In breast cancer patients, induced menopause was associated with weight gain (OR=4.12[1.76-9.67]), but no association was detected for tumor characteristics or treatments.

**Conclusion:**

This large prospective cohort provided original results on weight variation between before and after cancer diagnosis, highlighting different weight trajectories. Socio-demographic and economic factors appeared to influence the risk of weight gain, illustrating social inequalities in health.

## INTRODUCTION

More than 14 million of new cancer cases have been diagnosed in 2012 worldwide [1]. Due to the impact on physical, psychological and social functions of cancer itself and of secondary effects of anticancer therapies, malnutrition and weight loss are widespread in cancer patients [2–5]. A recent review showed that unintentional weight loss is a common issue in colorectal cancer patients [6]. In a recent French survey, 52% of patients with colorectal, pancreatic or gastric cancer experienced malnutrition [2]. Cancer-associated malnutrition has also been observed for non-digestive cancer locations [2, 4].

In contrast, weight gain has been described in many patients, especially in women diagnosed with breast cancer [7–13]. Weight gain has also been observed in prostate cancer patients who followed androgen deprivation therapy [14, 15]. Weight gain after breast and prostate cancer diagnosis is an important risk factor for poorer prognosis and recurrence [16–21], while weight management appears as a key component of tertiary prevention [22, 23]. In addition, in a recent meta-analysis, we showed that excess body weight at the diagnosis of a first breast cancer was associated with increased risk of second primary breast, endometrial and colorectal cancers [24]. Excess weight after diagnosis is also associated with higher all-cause mortality [21].

Since weight gain is a modifiable risk factor, some studies intended to identify its predictors in cancer patients [8–11, 13, 14]. Several factors have been suggested to influence weight gain after diagnosis including age [10, 11, 13, 14], educational level [11, 13], initial Body Mass Index (BMI) [11, 13], physical activity [25], energy intake [9, 10], smoking status [10], menopausal status and hormone receptors’ status for breast cancer [10], disease stage [10, 13] and cancer-related treatments [8, 12–14, 26–29]. However, these studies had limitations. They mostly focused on weight variation after cancer diagnosis and thus did not collect data regarding weight before diagnosis [2, 8, 14, 15, 25]. Few studies provided weight information before diagnosis, but all relied on pre-diagnosis anthropometric data collected retrospectively [9–11, 13]. To our knowledge, no study investigated weight change between before and after cancer diagnosis with anthropometric data collected prospectively, which would lower memory bias and substantially increase data quality. In addition, very few studies investigated a wide range of potential predictors of weight variation in the same dataset [10, 13, 25].

The aim of this study was to quantify weight variation (overall and by sex, cancer location and cancer prognosis) between before and after cancer diagnosis in a large cohort, relying on prospective anthropometric data. We also investigated socio-demographic, economic, lifestyle and clinical factors associated with moderate-to-severe weight gain.

## RESULTS

Characteristics of the study population are presented in Table 1 and Table 2. 68% of subjects were women. Mean age at diagnosis was 58±10.9 years. 40.1% had excess weight before cancer diagnosis. Mean time between inclusion in the cohort and diagnosis was 24.6±14.7 months. Main cancer locations were: breast (37%), prostate (15%), skin (10%) and colon-rectum (7%). Sex and age at diagnosis were similar between included and excluded cases (p=0.6 and 0.3 respectively) but breast and prostate cancers were more frequent in included cases (p=0.0007). Mean weight before (p=0.4) and respectively after (p=0.7) cancer diagnosis were similar between included and excluded cases with available weight data (375 excluded cases had weight data before diagnosis and 20 after respectively) (Appendix 1).

**Appendix 1:** Comparison between included and excluded cancer cases, NutriNet-Santé cohort, 2009-2015

	Included cases (N=1051)	Mean ± SD	Excluded cases (N=375)	Mean ± SD	p-value^1^
	N (%)		N (%)		
Age at diagnosis					
≤60years	506 (48.1 %)		176 (46.4%)		0.6
>60years	545 (51.9%)		203 (53.6%)		
Sex					
Male	339 (32.2%)		111 (29.3%)		0.3
Female	712 (67.8%)		268 (70.7%)		
Cancer locations					0.0007
Breast	385 (36.6%)		130 (34.3%)		
Prostate	162 (15.4%)		32 (8.4%)		
Skin	106 (10.1%)		32 (8.4)		
Colon-rectum	74 (7.0%)		34 (9.0%)		
Other	324 (30.8%)		151 (39.8%)		
Weight before diagnosis (kg)		69.96 ± 15.1		69.18 ± 15.1^2^	0.4
Weight after diagnosis (kg)		69.70 ± 15.3		71.55 ± 19.3^3^	0.7

^1^P-value of Student t-test for continuous variables and Chi-square test for qualitative variables.

^2^Available for 375 excluded cases.

^3^Available for 20 excluded cases.

**Table 1:** Sociodemographic, economic and lifestyle characteristics of first incident cancer cases of the NutriNet-Santé cohort, 2009-2015 (N=1051)^1^

	N	%	Mean	SD
Age at diagnosis (years)			58.5	10.9
Mean BMI before cancer diagnosis (kg/m^2^)			24.9	4.8
Mean BMI after cancer diagnosis (kg/m^2^)			24.8	4.9
Time between inclusion in the cohort and cancer diagnosis (months)			24.6	14.7
Sex				
Male	339	32.2		
Female	712	67.8		
Living area in Metropolitan France*^2^*				
Paris or Paris suburb	217	20.7		
North / North-East	152	14.5		
North-West	186	17.7		
Center	247	23.5		
South-East	135	12.8		
South-West	114	10.8		
Professionally active*^2^*				
No	580	44.8		
Yes	471	55.2		
Monthly income (€per household unit)*^2^*				
<1800	358	36.2		
1800-2700	288	29.1		
>2700	343	34.7		
Educational level*^2^*				
No higher education	461	43.9		
Under graduate	287	27.3		
Post graduate	298	28.8		
Smoking status				
Never smoker	930	88.5		
Former smoker (stopped at cancer diagnosis)	30	2.9		
Smoker after cancer diagnosis	91	8.6		
Excess weight*^3^* before cancer diagnosis				
No	630	59.9		
Yes	421	40.1		
Physical activity after cancer diagnosis^5^				
Physically active (moderate-to-intense)	650	78.1		
Low physical activity	182	21.9		
Energy intake variation before / after cancer diagnosis				
< -100 kcal/day	294	44.0		
[-100 - +100] kcal/day	158	23.6		
> +100 kcal/day	217	32.4		

^1^Multiple imputation was applied for missing data on covariates (N=382 for energy intake variation, N=219 for physical activity, N= 62 for monthly income, N=5 for educational level).

^2^At baseline, i.e. at inclusion in the NutriNet-Santé cohort study.

^3^BMI≥25kg/m^2^.

^4^From the validated IPAQ questionnaire.

**Table 2:** Cancer location and prognosis of first incident cancer cases of the NutriNet-Santé cohort, 2009-2015 (N=1051)^1^

	Breast^2^ (N=385)		Prostate^3^ (N=162)		Skin^4^ (N=106)		Colon-rectum^5^ (N=74)	
	N	%	N	%	N	%	N	%
Favorable prognosis	216	56.1	60	37.04	24	22.64	13	17.57
Poor prognosis	120	31.17	54	33.33	81	76.42	41	55.41
Missing data	49	12.73	48	29.63	1	0.94	20	27.03

^1^Multiple imputation was applied for missing data on covariates (N=49 for breast cancer, N=48 for prostate cancer, N=1 for skin cancer, N=20 for colon-rectum cancer), we have applied multiple imputation.

^2^Tumor size <2cm or node-negative or (tumor size <1cm and negative ER/PR receptors) = favorable prognosis; tumor size ≥2cm or node-positive or (tumor size ≥1cm and positive ER/PR receptors) = poor prognosis.

^3^PSA ≤20 ng/ml or Gleason ≤7 or cancer≤T2b= favorable prognosis; PSA >20 ng/ml or Gleason >7 or cancer >T2b = poor prognosis.

^4^Squamous cell carcinoma = favorable prognosis; melanoma = poor prognosis.

^5^(Cancer T1/T2 and node-negative) or no chemotherapy = favorable prognosis; (cancer T3/T4 and node-positive) or chemotherapy = poor prognosis.

The weight values before diagnosis were not statistically different (p=0.2). Similarly there was no difference between weight values after cancer diagnosis (p=0.9) (data not shown).

Weight variations between before and after cancer diagnosis are described in Table 3. Overall, no weight variation was observed (p=0.08) (relative difference=-0.22%, 95% confidence Interval=[-0.59; 0.16]). However, weight variation was significantly different between sex and cancer location (p=0.0003 and <0.0001 respectively): Weight loss was observed in men (p=0.0002, mean weight loss in men who lost weight=-3.54±4.39kg) and patients with colorectal cancer (p=0.001, mean weight loss in patients who lost weight=-3.94±4.40kg). In contrast, weight gain was observed in breast and skin cancer patients (p=0.04, mean weight gain=2.83±3.21kg and p=0.04, mean weight gain=2.96±2.75kg respectively). In breast and skin cancer patients, moderate-to-severe weight gain (15.6% for breast and 17.9% for skin cancers) was more frequent than moderate-to-severe weight loss (12.5% for breast and 8.5% for skin cancers).

**Table 3:** Weight variation between before and after cancer diagnosis, NutriNet-santé cohort 2009-2015 (N=1051)

	Weight variation between before and after cancer diagnosis						Moderate-to-severe weight variation (≥5% of initial weight)			
	Relative difference^1^	95% CI	p-value^2^	p-value for the difference between classes^3^	Weight loss in patients who lost weight	Weight gain in patients who gained weight	Moderate-to-severe weight loss (%)	No weight variation (%)	Moderate-to-severe weight gain (%)	p-value for the difference between classes^4^
					Mean ± SD	Mean ± SD				
Overall	-0.22	[-0.59; 0.16]	0.08		-3.37 ± 4.19	2.75 ± 3.08	15.2	72.3	12.5	
Sex				0.0003						0.005
Male	-1.25	[-1.87; -0.62]	0.0002		-3.54 ± 4.39	2.50 ± 3.49	15.3	77.9	6.8	
Female	0.27	[-0.19; 0.73]	0.6		-3.26 ± 4.06	2.84 ± 2.93	15.2	69.6	15.2	
Main cancer locations				<0.0001						<0.0001
Breast^5^	0.67	[0.09; 1.26]	0.04		- 2.90 ± 2.69	2.83 ± 3.21	12.5	71.9	15.6	
Favorable prognosis	0.75	[0.17; 1.72]	0.02		-2.90 ± 2.78	2.84 ± 3.43	10.3	75.4	14.3	
Poor prognosis	0.19	[-0.75; 1.14]	0.7		-3.04 ± 2.65	2.91 ± 2.66	16.3	65.7	18.0	
p-value between cancer prognosis^9^	0.2									
Prostate^6^	-0.18	[-0.87; 0.51]	0.5		-2.00 ± 2.74	2.13 ± 3.06	5.6	88.8	5.6	
Favorable prognosis	-0.57	[-1.48; 0.33]	0.2		-2.17 ± 2.68	1.57 ± 1.72	5.3	91.8	2.9	
Poor prognosis	0.16	[-1.03; 1.35]	0.8		-2.18 ± 3.02	2.52 ± 3.34	5.9	85.7	8.4	
p-value between cancer prognosis^9^	0.4									
Skin^7^	1.16	[0.18; 2.13]	0.04		-2.25 ± 2.22	2.96 ± 2.75	8.5	73.6	17.9	
Favorable prognosis	0.24	[-1.38; 1.87]	0.7		-2.12 ± 2.85	3.01 ± 2.30	16.7	70.8	12.5	
Poor prognosis	1.33	[0.15; 2.51]	0.04		-2.21 ± 1.98	3.0 ± 2.90	8.6	72.8	18.5	
p-value between cancer prognosis^9^	0.2									
Colon-rectum^8^	-2.21	[-3.55; -0.88]	0.001		-3.94 ± 4.40	1.93 ± 1.18	23.0	74.3	2.7	
Favorable prognosis	-0.54	[-2.99; 1.92]	0.7		-3.24 ± 3.66	2.36 ± 1.46	15.4	77.2	15.4	
Poor prognosis	-2.99	[-4.63; -1.34]	0.0004		-4.40 ± 4.34	1.40 ± 1.21	26.9	71.0	2.1	
p-value between cancer prognosis^9^	0.2									

^1^Relative difference = (mean weight after diagnosis mean weight before diagnosis) / mean weight before diagnosis*100.

^2^Paired Student's t-test comparing mean weight before and mean weight after cancer diagnosis.

^3^ANOVA test.

^4^Chi-square test.

^5^Tumor size <2cm or node-negative or (tumor size <1cm and negative ER/PR receptors) = favorable prognosis; tumor size ≥2cm or node-positive or (tumor size ≥1cm and positive ER/PR receptors) = poor prognosis.

^6^PSA ≤20 ng/ml or Gleason ≤7 or cancer ≤T2b= favorable prognosis; PSA >20 ng/ml or Gleason >7 or cancer >T2b = poor prognosis.

^7^Squamous cell carcinoma = favorable prognosis; Melanoma = poor prognosis.

^8^(Cancer T1/T2 and node-negative) or no chemotherapy = favorable prognosis; (CancerT3/T4 and node-positive) or chemotherapy = poor prognosis.

^9^Student's t-test.

Associations between moderate-to-severe weight gain and socio-demographic, economic and lifestyle factors are presented in Table 4. Women (OR_women/men_=1.75, 95%CI: [1.06-2.87], p=0.03), younger patients (OR_≤60years/>60_=2.44[1.51-3.70], p<0.0001), those with lower monthly income (OR_<1800€ per household unit vs >2700€_=1.30[1.01-1.72], p_trend_=0.007) lower educational level (OR_no higher education vs post-graduate_=1.32[1.03-2.70], p_trend_=0.03), those with excess weight before cancer diagnosis (OR_yes vs no_=1.64[1.12-2.42], p=0.01), who practiced lower physical activity after diagnosis (OR_low physical activity vs moderante-to-intense physical activity_=1.28 [1.01-1.64], p=0.04) and patients who stopped smoking after diagnosis (OR_stopped smoking vs never smoked_=4.31[1.99-9.35, p=0.005]) were more likely to gain weight. Similar trends were observed in breast cancer patients, although some results were not significant due to reduced statistical power (Table 4). No association was found among skin cancers (p>0.05) (Table 4).

**Table 4:** Socio-demographic, economic and lifestyle factors associated with moderate-to-severe weight gain^1^ between before and after cancer diagnosis, by unconditional logistic regression analyses, NutriNet-Santé cohort, 2009-2015 (N=1051)

	All cancers				Breast cancers				Skin cancers			
	No weight gain (n=920)	Weight gain (n=131)	Age and sex-adjusted		No weight gain (n=325)	Weight gain (n=60)	Age and sex-adjusted		No weight gain (n=87)	Weight gain (n=19)	Age and sex-adjusted	
			OR [95%CI]	p-value			OR [95%CI]	p-value			OR [95%CI]	p-value
Sex												
Male	316	23	1						23	6	1	
Female	604	108	1.75 [1.06-2.87]	0.03					64	13	1.60 [0.50-5.05]	0.4
Age at diagnosis												
≤60years	414	92	2.44 [1.51-3.70]	<0.0001	194	47	2.44 [1.27-4.76]	0.007	49	13	1.93 [0.63-5.92]	0.3
>60years	506	39	1		131	13	1		38	6	1	
Living area in Metropolitan France^2^												
Paris or Paris suburb	194	23	1		87	11	1		18	3	1	
North / North-East	128	24	1.53 [0.82-2.85]	0.2	36	14	2.93 [1.21-7.14]	0.02	17	1	0.30 [0.03-3.24]	0.1
North-West	165	21	1.08 [0.57-2.03]	0.6	52	7	1.04 [0.38-2.87]	0.3	15	6	2.12 [0.44-10.26]	0.2
Center	217	30	1.08 [0.60-1.94]	0.6	83	13	1.21 [0.51-2.87]	0.5	21	4	0.91 [0.17-4.80]	0.7
South-East	113	22	1.71 [0.90-3.25]	0.1	38	11	2.51 [0.99-6.37]	0.1	10	3	1.61 [0.26-10.05]	0.5
South-West	103	11	0.94 [0.44-2.03]	0.4	29	4	1.10 [0.32-3.75]	0.5	6	2	1.94 [0.23-16.27]	0.5
Professionally active^2^												
No	527	53	1		149	18	1		44	7	1	
Yes	393	78	1.03 [0.64-1.64]	0.9	176	42	1.31 [0.63-2.72]	0.5	43	12	1.65 [0.40-6.83]	0.5
Monthly income (€per household unit^2^				0.007^5^				0.8^5^				0.07^5^
<1800	301	57	1.30 [1.01-1.72]		109	16	0.86 [0.57-1.31]		26	11	2.29 [1.09-4.90]	
1800-2700	253	35	1.15 [0.86-1.54]		83	21	1.51 [0.99-2.27]		24	2	0.53 [0.18-1.55]	
>2700	317	26	1		115	16	1		31	4	1	
Educational level^2^				0.03^5^				0.09^5^				0.9^5^
No higher education	396	65	1.32 [1.03-2.70]		134	28	1.31 [0.87-1.92]		35	7	0.93 [0.46-1.70]	
Under graduate	251	36	0.96 [0.73-1.28]		93	19	1.12 [0.74-1.68]		21	6	1.17 [0.55-2.51]	
Post graduate	268	30	1		95	13	1		30	6	1	
Excess weight^3^ before cancer diagnosis												
No	559	71	1		226	34	1		60	12	1	
Yes	361	60	1.64 [1.12-2.42]	0.01	99	26	1.91 [1.08-3.39]	0.03	27	7	1.15 [0.39-3.40]	0.8
Physical activity after cancer diagnosis ^4^												
Physically active (moderate-to-intense)	582	68	1	0.04	218	34	1	0.2	54	13	1	0.7
Low physical activity	149	33	1.28 [1.01-1.64]		44	13	1.30 [0.89-1.89]		19	3	0.85 [0.42-1.75]	
Energy intake variation before / after cancer diagnosis				0.3^5^				0.9^5^				0.4^5^
< -100 kcal/day	255	39	1.16 [0.89-1.54]		90	17	1.03 [0.66-1.60]		28	7	1.42 [0.30-3.19]	
[-100 - +100] kcal/day	141	17	1		66	9	1		20	2	1	
> +100 kcal/day	198	19	0.90 [0.63-1.26]		51	10	1.09 [0.66-1.80]		19	2	0.84 [0.30-2.32]	
Smoking status												
Never smoker	831	99	1		291	47	1		79	16	1	
Former smoker (stopped at cancer diagnosis)	18	12	4.31 [1.99-9.35]	0.005	6	5	3.95 [1.14-13.67]	0.07	0	0	NA	NA
Smoker after cancer diagnosis	71	20	1.96 [1.13-3.40]	0.9	28	8	1.45 [0.61-3.43]	0.5	8	3	1.77 [0.41-7.64]	0.4

OR: odds ratio; CI: confidence interval; NA: not applicable.

^1^Weight gain ≥5% of the weight before diagnosis.

^2^At baseline, i.e. at inclusion in the NutriNet-Santé cohort study (before cancer diagnosis).

^3^BMI≥25 kg/m^2^.

^4^From the validated IPAQ questionnaire.

^5^p-trend.

No interaction was observed between cancer prognosis and socio-demographic, economic and lifestyle factors (all p>0.10) excepted for breast cancer cases for which an interaction was found between cancer prognosis and income (p=0.07). However, in stratified analysis, the association between monthly income and weight variation was non-statistically significant in both strata of cancer prognosis (p>0.05).

Results were similar when all factors of Table 4 were entered simultaneously into the model, except for monthly income and educational level which became non-statistically significant due to their strong correlation with other sociodemographic factors

The following clinical parameters were not associated with moderate-to-severe weight gain in female breast cancer patients: tumor size (p=0.9), lymph node status (p=0.6), tumor type (p=0.3), estrogen receptor (p=0.1), progesterone receptor (p=0.8), HER2 status (p=0.5), Ki67 (p=0.3), chemotherapy (p=0.5), hormone therapy (p=0.6), indicator of cancer severity (p=0.7), menopausal status (p=0.3) and cause of menopause (p=0.5). However women with induced menopause were more likely to experience moderate-to-severe weight gain (OR=4.12[1.76-9.67], p=0.001) (data not shown).

In sensitivity analyses, all results were similar when excluding subjects who had a second primary cancer or cancer recurrence during follow-up (n=37). Similar trends were observed when excluding anthropometric data collected less than 1 year or 2 years after cancer diagnosis, although some results became non-significant, due to loss of statistical power (n=855 and n=524 remaining subjects with available weight data respectively, data not shown).

## DISCUSSION

To our knowledge, this large cohort study was the first to investigate weight variations between before and after cancer diagnosis with anthropometric data collected prospectively, and a follow-up beginning in average 2y before diagnosis. Different weight trajectories were observed according to cancer locations. While weight loss was reported in many colorectal cancer patients, a substantial proportion of breast and skin cancer patients gained weight. Sociodemographic and economic factors appeared as important determinants of weight gain: patients with lower educational level or lower monthly income, women, and those aged below 60y were more likely to experience moderate-to-severe weight gain. Physical activity after diagnosis was associated with lower weight gain. In contrast, initial excess weight and smoking cessation after cancer diagnosis were strongly associated with weight gain. In breast cancer patients, induced menopause was also strongly associated with weight gain, while tumor characteristics and treatments did not appear as major predictors of weight gain in this study.

### Weight variations according to cancer location

A substantial proportion (23%) of colorectal cancer patients experienced moderate-to-severe weight loss in this study, consistent with previous literature [6, 36]. This may be due to the fact that digestive function is directly affected in this pathology. A recent review showed that this weight loss has devastating effects on patients’ self-image, quality of life, and survival [6].

In contrast, 16% of breast cancer cases experienced moderate-to-severe weight gain in our cohort. Consistently, many studies observed weight gain after female breast cancer diagnosis in Western countries [8–13, 28, 37], with varying proportions of patients gaining weight. In a recent French survey [8], 60% of the cohort gained weight after breast diagnosis. A systematic review published in 2011 indicated that 50-96% of women experienced weight gain during breast cancer treatment [12]. However, most of these studies considered cancer diagnosis as baseline, while this potentially corresponds to a period of strong weight fluctuation [11]. Indeed, it has been shown that patients who gained weight after cancer in a long-term basis were likely to first lose weight just before/around diagnosis [11]. Thus, in the present study, we excluded anthropometric data collected 3 months before and 6 months after (12 and 24 months in sensitivity analyses) cancer diagnosis, in order to focus on stabilized periods.

In our study, we did not observe any association between clinical characteristics and treatments of breast tumors and the risk of weight gain. These results should be considered with caution since statistical power was limited for several parameters. However, these results were consistent with previous studies that showed no association between tumor stage [9, 11] or hormone receptor status [13] and weight gain after breast cancer. In contrast, other studies suggested that mixed (+/-) receptor status and more advanced tumor stage could increase the risk of weight gain [10, 13, 37]. Concerning the association between cancer treatments and weight change, mixed results have been reported [8, 12, 13]. In particular, adjuvant therapy has been linked to a higher risk of weight gain in some studies [12, 13, 27] but not in others [28, 29, 38].

Breast cancer women with induced menopause (caused by ablation of the uterus and/or both ovaries, by radiotherapy or by chemotherapy) had a drastically increased risk of weight gain. This phenomenon, already known in the general population [39], has been described in the specific context of breast cancer in an American study where 70% of women being treated with ovarian suppression reported moderate-to-severe weight gain [40].

A substantial proportion (18%) of skin cancer patients also experienced moderate-to-severe weight gain in our study. To our knowledge, this has not been investigated in previous literature and deserves confirmation in future studies. One of the explanations may be that patients diagnosed with skin cancer receive medical advice to avoid sun exposure, which could limit their practice of outdoor physical activity.

### Weight variations according to socio-demographic, economic and lifestyle characteristics

This study suggests that sociodemographic and economic factors are associated with weight gain between before and after cancer diagnosis. Subjects with lower educational level were more likely to gain weight, consistent with a previous study on breast cancer patients [13]. Lower monthly income was also associated with higher risk of weight gain in our study, but not independently from educational level. In developed countries, education is generally inversely associated with overweight and obesity via adult socioeconomic status (e.g. income and occupation), but also via health literacy, health behaviors, and sense of control and empowerment [41]. The fact that women were more likely to gain weight than men after cancer diagnosis is probably driven by our results regarding weight gain in breast cancer patients. In our study, we found that subjects who were aged ≤60 at cancer diagnosis were more likely to gain weight than their older counterparts, as shown in others studies [10, 13, 14, 38]. This may be due to the fact that older patients are more prone to malnutrition and subsequent weight loss [42].

Consistent with previous findings [43], patients already overweight before diagnosis were more likely to gain weight, suggesting that weight management issues track after cancer onset. Interestingly, we showed that smoking cessation after diagnosis was strongly associated with weight gain. This phenomenon is well described in the general population [44, 45], but is of particular importance in cancer patients, who are strongly advised to stop smoking after diagnosis. Patients who were smoking after cancer diagnosis were also at higher risk of weight gain compared to never smoker consistent with another study [10]. Moderate-to-intense physical activity after diagnosis was associated with lower weight gain in sex and age-adjusted models. This point is debated in the literature, some studies suggesting an impact of decreasing physical activity on weight gain [25, 46], while a recent review in prostate cancer patients concluded that exercise alone did not lead to weight loss [47].

Strengths of this study pertained to a large population-based cohort with incident cancer cases, prospective anthropometric data collected before and after cancer diagnosis and detailed information on socio-demographic, economic, lifestyle and clinical parameters.

Several limitations should be acknowledged. First, caution is needed in extrapolating our results to all French cancer cases, since the NutriNet-Santé study involved volunteers who accepted to participate in a survey on nutrition and health. Indeed, compared to national estimates, the NutriNet-Santé study included more women and individuals belonging to higher socio-professional categories, as often observed in population-based observational cohorts [48]. Besides, despite lower incidence rates, main cancer locations represented in our cohort were same as in the general population (breast, prostate, colorectal) [49] was similar in our cohort. As in all observational cohorts some cancer cases may have been missed. We hypothesize that such cancer cases may have had a poorer prognosis; therefore this study may have overestimated the proportion of cancers with better prognosis. Moreover, a number of cancer cases were excluded due to missing weight data before or after diagnosis and some of their characteristics (cancer location) differed from those of included cases. Furthermore, even if we have tested all relevant factors available in our cohort; some others (such as psychological factors) were not taken into account. Next, weight and height were self-reported, thus classification bias could not be excluded. However, in a previous validation study on 199 subjects, we showed that self-reported weight and height data from the NutriNet-Santé web-based study were valid and strongly correlated with anthropometric data measured by study staff [50]. Finally, while medical records were available for validation of all cancer cases, exhaustive clinical data were not systematically recorded in these files. These missing values for clinical factors in some patients may have impaired our ability to detect some of the hypothesized associations.

In conclusion, this large cohort of cancer patients provided original prospective results on weight variation between before and after cancer diagnosis, highlighting different weight trajectories. While weight loss was widespread after cancer diagnosis (especially for digestive cancers), a substantial proportion of cancer survivors (especially breast cancers) experienced moderate-to-severe weight gain. More than tumor characteristics or treatments, socio-demographic and economic factors appeared to influence the risk of weight gain, illustrating social inequalities in health (higher risk in patients with lower educational level or lower income). Since excess weight has been recognized as a key modifiable risk factor for recurrence and second cancer, efforts are needed to encourage cancer survivors to achieve or maintain healthy body weight [22]. Results of the present study provide insights to identify and target the sub-groups of patients who are more specifically at-risk of moderate-to-severe weight gain.

## MATERIALS AND METHODS

### The NutriNet-Santé cohort

The NutriNet-Santé study is a large web-based cohort launched in May 2009 to evaluate the determinants of eating behavior and the relationships between nutrition and chronic disease risk in the French general population. Participants are recruited by vast multimedia campaigns. Inclusion criteria are: age ≥ 18 y and access to the Internet. Participants register and are followed-up online using a dedicated website (www.etude-nutrinet-sante.fr). The recruitment is still ongoing. The NutriNet-Santé study was approved by the Institutional Review Board of the French Institute for Health and Medical Research (IRB Inserm n°0000388FWA00005831) and the “Commission Nationale de l’Informatique et des Libertés” (CNIL n°908450 and n°909216).

### Data collection

At baseline and each year thereafter, participants completed a set of five self-administered web-based questionnaires on socio-demographic and lifestyle characteristics (sex, age, living area, employment status, monthly income per household unit, educational level, and smoking status), anthropometrics (weight and height), dietary intake (3 non-consecutive 24-h dietary records), physical activity (validated IPAQ questionnaire [30]), and health status. All these instruments have been tested against traditional assessment methods (paper-and-pencil questionnaires or interview by a dietitian) [31–33]. Intermediate self-administered questionnaires were also used to collect weight, height and dietary data every 6 months. In women, information regarding menopause (date, natural or induced, and hormonal treatment) was obtained from health questionnaires.

### Case ascertainment

Participants self-declared any cancer diagnosis during follow-up through regular questionnaires every 6 months and a permanent web-interface. Pathological reports were used by an independent physician expert committee for validation. All cancer cases were validated and classified using the International Chronic Diseases Classification, 10th Revision, Clinical Modification (ICD-10) [34]. All first incident cancers were considered as cases in this study, except basal cell carcinoma (not considered as cancer).

For the four main cancer locations represented in this study (breast, prostate, skin, colon-rectum), tumor characteristics and treatments were extracted from medical records: for breast cancer: tumor size, lymph node status, tumor type (invasive or *in situ*), estrogen and progesterone receptor status, HER2 status, Ki67 and treatment (chemotherapy and/or hormone therapy); for prostate cancer: tumor size, lymph node status, PSA, Gleason score and treatment (chemotherapy, radiotherapy and/or hormone therapy); for skin cancer: melanoma: Breslow index and Clark level and squamous cell carcinoma: type of tumor (invasive or *in situ*); for colorectal cancer: tumor size, lymph node status and treatment. Given the low number of advanced stages for each tumor location, using TNM/UICC stages was not discriminating, thus, patients were classified into two categories (favorable prognosis/poor prognosis) according to clinically pertinent factors, as described in footnotes to Table 1.

### Statistical analysis

From the 1704 cancer cases diagnosed in the NutriNet-Santé study between May 2009 and June 2015 and with at least 6months of follow-up after diagnosis, 1426 cases were first incident cancers. We excluded 350 patients with missing BMI before or after cancer diagnosis and 25 pregnant women, leaving 1051 cancer cases for analysis.

For each subject, mean weight before (respectively after) diagnosis was calculated as the average of all weight data before (respectively after) cancer diagnosis. Weight data declared in the timeframe [-3 months before; +6 months after] cancer diagnosis were excluded from the calculation, in order to focus on stable periods. Relative difference was calculated as (mean weight after diagnosis – mean weight before diagnosis)/mean weight before diagnosis*100. BMI was calculated as weight(kg)/height(m)^2^. Similarly, variation in mean daily energy intake between before and after diagnosis was calculated. Baseline socio-demographic data were used for the present analysis. Multiple imputations were applied for all covariates with missing data (monthly incomes, educational level, physical activity after cancer diagnosis, energy intake variation before / after cancer diagnosis and cancer prognosis) [35].

Weight before and after cancer diagnosis was compared by paired Student's t-test. These variations were compared between sex, cancer location and cancer prognosis by ANOVA. Weight variations were classified into 3 categories: “moderate-to-severe weight loss” (weight loss ≥5% of initial weight), “moderate-to-severe weight gain” (weight gain ≥5% of initial weight), and “no major weight variation” otherwise. Weight evolutions were compared between sex, cancer location and cancer prognostic by Chi-square tests.

Age and sex-adjusted unconditional logistic regression analyses were used to investigate the associations between moderate-to-severe weight gain and socio-demographic and economic factors (sex, age, living area, professional activity, monthly income, and educational level), and lifestyle factors (excess weight before cancer diagnosis, variation of daily energy intake, variation of smoking status before/after diagnosis, and physical activity after diagnosis). This analysis was conducted for overall cancer and breast cancer specifically (main cancer location in the cohort). We also tested two-way interactions between all these sociodemographic, economic and lifestyle parameters, and cancer prognosis.

In breast cancer cases, unconditional logistic regression analyses adjusted for above listed factors were used to investigate the associations between moderate-to-severe weight gain and tumor characteristics and treatments, and menopausal status and cause.

Sensitivity analyses were performed by excluding subjects with cancer recurrence or second primary cancer during follow-up, and by excluding anthropometric data collected less than 1 year or 2 years after cancer diagnosis.

P-value<0.05 was considered statistically significant. All tests were two-sided. Analyses were carried out with SAS 9.3 (SAS Institute Inc, Cary, NC, USA).

## Abbreviations

BMI: body mass index
OR: odds ratio
CI: confidence interval
